# Effects of coconut oil on glycemia, inflammation, and urogenital microbial parameters in female Ossabaw mini-pigs

**DOI:** 10.1371/journal.pone.0179542

**Published:** 2017-07-13

**Authors:** Annie E. Newell-Fugate, Katherine Lenz, Cassandra Skenandore, Romana A. Nowak, Bryan A. White, Andrea Braundmeier-Fleming

**Affiliations:** 1 Department of Veterinary Physiology and Pharmacology, Texas A&M University, College Station, Texas, United States of America; 2 Department of Animal Sciences, University of Illinois at Urbana-Champaign, Urbana, Illinois, United States of America; 3 Department of Medical Microbiology and Immunology, Southern Illinois University School of Medicine, Springfield, Illinois, United States of America; 4 Carl R. Woese Institute of Genomic Biology, University of Illinois at Urbana-Champaign, Urbana, Illinois, United States of America; 5 Department of Obstetrics and Gynecology, Southern Illinois University School of Medicine, Springfield, Illinois, United States of America; INIA, SPAIN

## Abstract

Forty percent of American women are obese and at risk for type II diabetes, impaired immune function, and altered microbiome diversity, thus impacting overall health. We investigated whether obesity induced by an excess calorie, high fat diet containing hydrogenated fats, fructose, and coconut oil (HFD) altered glucose homeostasis, peripheral immunity, and urogenital microbial dynamics. We hypothesized that HFD would cause hyperglycemia, increase peripheral inflammation, and alter urogenital microbiota to favor bacterial taxonomy associated with inflammation. We utilized female Ossabaw mini-pigs to model a ‘thrifty’ metabolic phenotype associated with increased white adipose tissue mass. Pigs were fed HFD (~4570 kcal/pig/day) or lean (~2000 kcal/pig/day) diet for a total of 9 estrous cycles (~6 months). To determine the effect of cycle stage on cytokines and the microbiome, animals had samples collected during cycles 7 and 9 on certain days of the cycle: D1, 4, 8, 12, 16, 18. Vaginal swabs or cervical flushes assessed urogenital microbiota. Systemic fatty acids, insulin, glucose, and cytokines were analyzed. Pig weights and morphometric measurements were taken weekly. Obese pigs had increased body weight, length, heart and belly girth but similar glucose concentrations. Obese pigs had decreased cytokine levels (IL-1β, TNF-α, IL-4, IL-10), arachidonic acid and plasma insulin, but increased levels of vaccenic acid. Obese pigs had greater urogenital bacterial diversity, including several taxa known for anti-inflammatory properties. Overall, induction of obesity did not induce inflammation but shifted the microbial communities within the urogenital tract to an anti-inflammatory phenotype. We postulate that the coconut oil in the HFD oil may have supported normal glucose homeostasis and modulated the immune response, possibly through regulation of microbial community dynamics and fatty acid metabolism. This animal model holds promise for the study of how different types of obesity and high fat diets may affect metabolism, immune phenotype, and microbial dynamics.

## Introduction

Obesity is at epidemic levels, particularly in western cultures such as the United States, where recent epidemiologic statistics indicate that 40% of American women are obese [1]. Obesity is associated with the development of metabolic syndrome, and can lead to additional health issues such as type II diabetes, cardiovascular disease, atherosclerosis, and cancer. As such, obesity and its sequelae cause an estimated financial burden on the United States healthcare system of $75 billion per year [2]. Metabolic disturbances associated with obesity such as hyperlipidemia, dyslipidemia and insulin resistance can be characterized by a loss of immune homeostasis within the body, further perpetuating an environment of low-grade chronic inflammation [3]. In fact, the development of insulin resistance and type II diabetes, even independent from obesity, can be predicted by an increase in inflammatory markers like interleukin 6 (IL-6) and C-reactive protein (CRP) [4, 5]. Furthermore, excess adipose tissue results in increased numbers of macrophages within adipose tissue, which produce several pro-inflammatory cytokines, including tumor necrosis factor alpha (TNF-α), interleukin 1 (IL-1), and IL-6 [6]. These excess cytokines establish a positive-feedback loop through their increased receptor activation, resulting in chronic inflammation.

Early diagnosis of immunological and microbial community dysregulation could aid in early detection of obesity-associated inflammation and thus diminished overall health. Several studies have demonstrated the role of the microbiome in the development of the host immune system, as mice lacking microbes in the gastrointestinal tract fail to undergo immune cell maturation and have a dysregulated immune response [7, 8]. Disruptions in commensal bacterial species that comprise the microbiome can lead to both metabolic and immune diseases, emphasizing the symbiosis between these two systems necessary for physiological homeostasis in the host. Observation of the types and numbers of bacterial species that populate the urogenital mucosa permits assessment of immune balance in the female reproductive tract. Bacterial communities associated with a ‘healthy’ urogenital microbial profile have protective and immunologically beneficial roles in the reproductive tract. A shift of microbial species may be indicative of immune dysfunction and possibly systemic inflammation.

Previous studies have utilized the Ossabaw pig as an ideal model for the study of obesity and its effects on reproductive function in females. Ossabaw pigs have a loss of function mutation in the Val199→ Ile region of the PRKAG3 gene, which is the γ^3^ isoform of AMP-activated protein kinase. Upon activation, this kinase phosphorylates enzymes involved in insulin signaling and cholesterol and fatty acid metabolism, and is therefore associated with accumulation of increased subcutaneous, visceral, and intramuscular fat and a ‘thrifty’ phenotype [9]. These pigs serve as an excellent model for obesity as they naturally develop a condition that mimics metabolic syndrome in humans when fed an excess-calorie, high-fat/cholesterol/fructose diet, including visceral obesity, glucose intolerance, insulin resistance, dyslipidemia, and hypertension [9–14].

The goals for this study utilizing female Ossabaw pigs as a model of obesity were to: 1) measure body weight and measurements, glucose and insulin concentrations, and plasma free fatty acid composition to assess metabolic function, 2) assess systemic immune function through analysis of serum cytokine levels, and 3) identify bacterial communities in the urogenital tract associated with obesity. Specifically, we hypothesized that obesity induced by a high fat diet rich in hydrogenated fats and a plant-derived saturated fat, coconut oil, would cause hyperglycemia, hyperinsulinemia, decrease the immune protection of the urogenital microbial community profile, and favor systemic inflammation.

## Materials and methods

### Animal husbandry and diet

All experimental procedures performed on animals were done by a protocol approved specifically by the University of Illinois at Urbana-Champaign Institutional Animal Care and Use Committee (IACUC; protocol # 11114), and followed the guidelines as described in the *Guide for the Care and Use of Laboratory Animals* [15]. Five nulliparous, sexually mature female Ossabaw pigs were acquired from the Comparative Medicine Program of Indiana University School of Medicine and Purdue University (West Lafayette, IN, USA). The obese diet utilized in this study was comprised of a base pelleted pig feed (5L1G; custom formulated by Purina TestDiet, Inc., Richmond, IN, USA) supplemented with hydrogenated soybean oil (8.4%), coconut oil (4.7%), high fructose corn syrup (5.0%), cholesterol (2.0%), and sodium cholate (0.7%) by weight. The lean diet fed in this study was the Rund Diet (University of Illinois at Urbana-Champaign, Urbana, IL, USA) comprised of corn (57.5%) and soy (40.0%) supplemented with vitamins and minerals. During the six-month study period, lean pigs (n = 2) were fed approximately 2000 kcal of the pelleted lean diet per pig per day, and obese pigs (n = 3) were fed approximately 4570 kcal of obese diet per pig per day. Water was available *ad* libitum. The obese phenotype was induced over three months of dietary treatment (induction phase), following which the diets continued to be fed at the same levels for an additional 3 months (maintenance phase). During the study, pigs were housed individually to ensure exact dietary dosing and were on a 12:12-hour light:dark cycle. Euthanasia was performed by sodium pentobarbital overdose following administration of 1 ml/45 kg body weight of a combination of telazol + ketamine + xylazine. Every effort was made to minimize animal suffering.

### Sample collection

The design of this study used repetitive sampling from each individual, which allowed for each animal to serve as their own internal baseline control, and thus generate substantial, significant data with a limited sample size. Heat detection was conducted in the presence of a boar and was used to measure estrous cycle length to determine sampling time periods. Jugular blood collection, vaginal swabs, and cervical flushes were performed on day 1 (estrus, standing heat) and on day 8 (mid-luteal phase) of the estrous cycle for the first six continuous estrous cycles (induction phase). Samples were also collected on cycles 7 and 9 (maintenance phase), on days 1 (estrus), 4, 8, 12, 16, 18 or until pigs stood in heat again. A Panepinto low stress sling restraint system was used on all pigs for the purposes of restraint for blood collection [16]. Vaginal swabs were collected by inserting a sterile Dacron swab into the vagina (to a depth of < 1 inch); the swab was then rotated for 360° while scraping the vaginal wall for no more than 10 seconds, resulting in minimal discomfort to the animal. Once collected, the swab tip was placed into a plastic tube containing 1 mL sterile saline. The tube was tightly capped and placed immediately on dry ice. Cervical flushings were collected by sanitizing the vulva with betadine and 70% ethanol followed by insertion of a sterile catheter through the vagina and the cervix. After catheter placement, using sterile technique, a syringe filled with 12 ml of sterile saline was placed on the catheter and the saline was pushed into the cervix. A plunger was used to move the saline back and forth to get a good flush before withdrawing the catheter and syringe containing the cervical flushing. Cervical flushings were placed immediately on dry ice. Vaginal swabs and cervical flushing were stored at -80°C until processed for DNA extraction.

### Metabolic parameters

Physical measurements were taken weekly, including weight, crown to rump length, heart girth, belly girth, and height. Blood glucose concentrations were measured on D1, 4, 8, 12, 16, 18 for cycles 7 and 9 from blood obtained by small ear pricks. Glucose testing was performed with the Precision Xtra blood glucose and ketone monitoring system (Abbott Diabetes Care, Alameda, CA, USA), which has been validated for use in the Ossabaw pig [14]. Free fatty acid analysis was performed on plasma collected on D1 and D8 from cycle 1 and cycle 7. Plasma insulin was assessed on D1 and D8 from cycle 1, 6, 7, and 9 using the Porcine Insulin RIA (EMD Millipore, Billerica, MA, USA). As a proxy for insulin sensitivity in each diet treatment group, a homeostatic model of assessment for insulin resistance (HOMA-IR = (glucose*insulin/405)) was calculated prior to diet treatment and during the maintenance phase (cycles 6,7,9) of diet treatment for each treatment group. For the assessment of free fatty acids, one ml plasma was extracted in chloroform:methanol (2:1, v/v) and fatty acid methyl esters (FAME) were prepared as described by Morrison and Smith [17], modified to include an additional saponification step [18]. FAME were analyzed with a Varian gas chromatograph (model CP-3800 fixed with a CP-8200 autosampler, Varian Inc., Walnut Creek, CA, USA). Separation of FAME was accomplished on a fused silica capillary column CP-Sil88 [100m x 0.25 mm (i.d.)] (Chrompack Inc., Middleburg, The Netherlands), with hydrogen as the carrier gas. Column oven temperature was increased from 150 to 160°C at 1°C per min, from 160° to 167°C at 0.2°C per min, from 167 to 225°C at 1.5°C per min, and then held at 225°C for 16 min. The injector and detector were maintained at 250°C. Total run time was 60 min. Individual fatty acids were identified using genuine external standards (Nu-Chek Prep, Inc., Elysian, MN, USA). Insulin and fatty acid levels were measured and compared within each individual and also across all pigs to minimize variability and to assess the effect of the diet over time.

### Immune phenotype

The levels of seven cytokines (Interleukin 1-beta (IL-1β), Interleukin 10 (IL-10), Interferon alpha (IFN-α), Interferon gamma (IFN-γ), Tumor necrosis Factor alpha (TNF-α), Interleukin 4 (IL-4), and Interleukin 8 (IL-8)) in serum from days 1, 8, 12, 16, and 18 of cycles 1, 7 and 9 of each pig were analyzed simultaneously using the Swine Cytokine Magnetic 7-Plex Panel assay (Novex^®^, Life Technologies Ltd., UK). Analysis of the assay was performed on a Luminex^®^100/200^™^ in the Research Services Core at Southern Illinois University School of Medicine. Cytokine levels were measured and compared within each individual and also across all pigs to minimize variability. Samples were taken at multiple time points during cycles 7 and 9 (maintenance) to gauge whether reproductive cyclicity had an effect on the levels of each cytokine.

### Microbial assessment

DNA was extracted from the vaginal swabs and cervical flushings of all pigs using the PowerSoil^®^ DNA Isolation Kit (Mo Bio Laboratories, Inc., Carlsbad, CA, USA) according to the manufacturer’s instructions. After extraction the DNA stock concentration was measured using a NanoDrop-2000 spectrophotometer (Thermo Scientific, Inc., Wilmington, DE, USA), with samples ranging from 12.6 to 510 ng/μl.

A 584 bp fragment of the hypervariable V3-V5 region of the 16S rRNA gene was amplified by a polymerase chain reaction (PCR) as follows: 25 μl Kapa HiFi (Kapa Biosystems, Woburn, MA, USA), 25 μM forward primer, 25 μM reverse primer, 50 ng of DNA, and molecular grade water to reach a final volume of 50 μl per reaction. The 357F forward primer was used (read 1: 5’TATGGTAATTGTCCTACGGGAGGCAGCAG3’; read 2: 5’AGTCAGTCAGCCCCGTCAATTCMTTTRAGT3’; index: 5’ACTYAAAKGAATTGACGGGGCTGACTGACT3’). The universal reverse primer was 926R (5’CCGTCAATTCMTTTRAGT3’). One of 96 specific barcodes consisting of 12 base pairs was also added to the reverse primer. These unique barcodes serve as a genetic ID for each sample to be sequenced. The PCR cycle was as follows: 45 seconds at 98°C followed by 25 cycles of 15 seconds at 98°C, 30 seconds at 65°C, and 30 seconds at 72°C for denaturation, and then 2 minutes at 72°C and hold at 4°C for final extension, as described in the HMP [19]. The PCR products were then run on an agarose gel, where it was determined that a nested PCR amplification was required. Following the nested PCR, the products were then purified using the GeneJET PCR Purification Kit (Thermo Scientific, Inc., Wilmington, DE, USA). Final DNA product concentrations were measured by the NanoDrop-2000 spectrophotometer and were between 36.8 and 226.8 ng/μl. Samples were then pooled together by mass (15 μg), with one pool consisting of samples containing reverse primer barcodes 1–96. Total pool concentration was measured by Qubit 2.0 Fluorometer (Invitrogen, Life Technologies, Carlsbad, CA, USA). All pools were then sent to the University of Illinois at Urbana-Champaign to be sequenced utilizing a high-throughput platform (MiSeq; Illumina Inc., San Diego, CA,USA). Approximately 87,000,000 total sequence reads with 50,000 reads/sample were obtained, including both dominant and poorly-represented taxa of the urogenital microbiome.

Sequences were analyzed using the QIIME pipeline [20, 21], removing poor quality incomplete sequences. Sequences were then fed through the Ribosomal Database Project in order to obtain the taxa calls that were used in later correlation analysis. This bioinformatics pipeline allows operational taxonomical unit (OTU) assignments followed by microbial community analyses including sequence alignment, phylogenetic trees, phylogenetic- and taxon-based analysis of diversity, and network analysis, as well as Unifrac analysis for clustering into OTUs, generating rarefaction curves and calculating the species diversity.

### Statistical analysis

As a means to control for individual variability, free fatty acid concentrations on cycle 1 day 1 (induction) and cycle 7 day 1 were used to calculate the change in free fatty acids within each animal over time. Insulin concentrations on cycle 1 days 1 and 8 and cycles 6, 7, and 9 days 1 and 8 were used to calculate the change in insulin within each animal over time while on their respective diet. This same comparison was made for the HOMA-IR parameter across treatment groups. This type of calculation was not performed on glucose concentrations, because glucose concentration initially was affected by the stress of handling, which resulted in glucose levels being high in both treatment groups at the onset of the study.

All metabolic data were assessed using PROC GLM (SAS, Inc, Cary, NC, USA) for normality and transformed as needed for non-normal samples prior to analysis. A repeated measures in time ANOVA was conducted (SAS, Inc., Cary, NC, USA). The Akaike criterion was used to determine the best fit covariance matrix, which was the autoregressive matrix, and all parameters were run using this matrix.

Cytokine concentrations at each time point within each animal were normalized to the animal’s own estrous cycle 1, day 1 (induction), meaning that statistical differences for each individual were calculated as the change between sampling timepoints (i.e. cycle 1, day 1 vs. cycle 7, day 1), and then this difference was compared between all individuals. All samples were normalized to the background fluorescent intensity (no serum control) from the unknown sample fluorescent intensity, which was calculated from the standard curves provided in the assay kit, which yielded average concentrations across treatment groups. A Wilcoxin signed rank test was conducted to determine significance between treatment groups throughout the treatment timeline.

To determine the relationship between microbiomes, data were visualized with 2D or 3D non-metric multidimensional scaling (NMDS) plots, which were constructed by assigning a non-parametric monotonic relationship to the Bray-Curtis dissimilarities using isotonic regression to produce a two- or three-axis representation of the relationships between data. Subsequently, a permutational MANOVA test was used to assess the factors driving bacterial community composition. Bray-Curtis distance analysis, coordinate, and statistical analyses used to cluster microbial profiles were determined as previously described [22].

All results are presented as the least squared mean ± standard error of the mean. In all statistical tests, *p*<0.05 was the criterion to indicate statistical significance unless otherwise noted.

## Results

### Metabolic parameters

Weight (lean, 57.8 ± 5.4 kg; obese, 73.5 ± 1.8 kg), crown to rump length (lean, 84.8 ± 1.3 cm; obese, 92.7 ± 1.3 cm), heart girth (lean, 85.6 ± 1.3 cm; obese, 99.3 ± 1.3 cm), and belly girth (lean, 87.1 ± 1.3 cm; obese, 102.9 ± 1.3 cm) were all significantly (*P* ≤ 0.05) greater in obese pigs compared with lean (Table 1). Height (lean, 58.6 ± 1.1 cm; obese, 58.5 ± 1.5 cm) was not different between lean and obese pigs (Table 1). No differences were detected in glucose concentrations between the two treatment groups (Table 2). Obese pigs developed lower plasma insulin concentrations while on diet compared with lean pigs, which developed higher plasma insulin concentrations while on diet (Table 2). However, there was no significant difference between the change in HOMA-IR between the two diet groups (Table 2). Pigs that consumed the high fat diet developed decreased levels of arachidonic acid (*P* = 0.04) while on the diet as compared with lean pigs (Table 2). There was a trend for obese pigs to develop increased levels of cis-vaccenic acid (*P* = 0.10) while on the obese diet as compared to lean pigs (Table 2).

**Table 1:** Morphometric measurements for lean and obese Ossabaw pigs.

Measurement	Lean	Obese	*P*-value
Crown to Rump Length (cm)	84.8 ± 1.3	92.7 ± 1.3	<0.0001
Heart Girth (cm)	85.6 ± 1.3	99.3 ± 1.3	<0.0001
Belly Girth (cm)	87.1 ± 1.3	102.9 ± 1.3	<0.001
Height (cm)	58.6 ± 1.1	58.5 ± 1.5	>0.05
Body weight (kg)	57.8 ± 5.4	73.5 ± 1.8	<0.0001

**Table 2:** Average glucose concentration and change in insulin, HOMA-IR, and free fatty acids from beginning to end of trial for lean and obese pigs.

Common name	Lipid Numbers	Lean	Obese	*P*-value
Glucose (mg/dL)	N/A	64.0 ± 2.1	66.2 ± 1.7	0.38
**Insulin (μU/ml)**	**N/A**	**3.1 ± 1.9**	**-7.9 ± 3.1**	**0.006**
HOMA-IR	N/A	-0.401 ± 0.5781	0.265 ± 0.425	0.5
Myristic acid	C14:0	-0.20 ± 0.13	-0.19 ± 0.10	0.95
Myristoleic acid	C14:1	0.00 ± 0.25	0.40 ± 0.21	0.3
Palmitic acid	C16:0	-13.12 ± 5.09	-0.48 ± 4.16	0.15
Palmitoleic acid	C16:1	0.00 ± 0.30	0.64 ± 0.24	0.19
Stearic acid	C18:0	-0.46 ± 4.00	-2.36 ± 3.27	0.74
Oleic acid	C18:1	3.99 ± 7.77	1.83 ± 6.34	0.84
**cis-Vaccenic acid**	**C18:1n-7**	**-0.23 ± 0.45**	**1.12 ± 0.37**	**0.10**
Linoleic acid	C18:2	3.14 ± 3.31	0.76 ± 2.70	0.62
γ-Linolenic acid	C18:3	0.00 ± 0.11	0.17 ± 0.09	0.33
Arachidic acid	C20:0	0.00 ± 0.06	0.02 ± 0.05	0.81
Paullinic acid	C20:1	0.00 ± 0.00	0.00 ± 0.00	1
Eicosadienoic acid	C20:2	-0.03 ± 0.10	0.07 ± 0.08	0.53
**Arachidonic acid**	**C20:4**	**6.42 ± 2.62**	**-5.61 ± 2.14**	**0.04**
Eicosatrienoic acid	C20:3n-3	0.00 ± 0.00	0.00 ± 0.00	1
Behenic acid	C22:0	0.00 ± 1.64	1.64 ±1.34	0.5
Eicosahexaenic acid	C22:1+C20:5	0.00 ± 0.00	0.00 ± 0.00	1
Lignoceric acid	C24:0	0.47 ± 0.36	0.03 ± 0.29	0.41
Nervonic acid	C24:1	0.13 ± 0.52	-0.03 ± 0.43	0.83
Docosahexaenoic acid	C22:6	-0.11 ± 0.13	0.24 ± 0.10	0.12

### Immune phenotype

**Fig 1:**
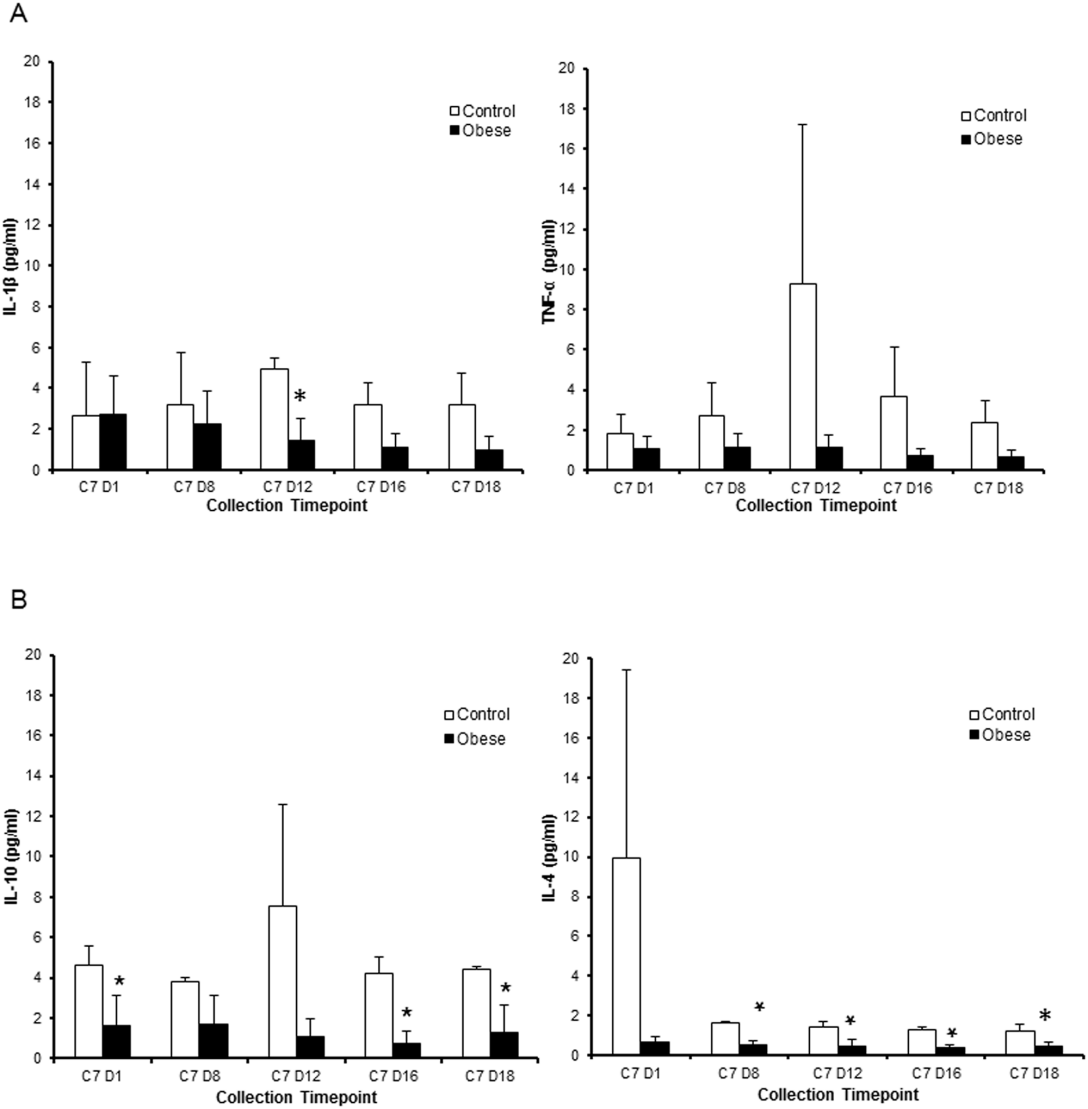
Serum cytokine measurements throughout cycle 7 (dietary maintenance) in obese and lean pigs. (A) Levels of pro-inflammatory cytokines IL-1β and TNF-α. (B) Levels of anti-inflammatory cytokines IL-10 and IL-4. Results are expressed as least squared mean fluorescent intensity ± SEM (obese, *n* = 3; control, *n* = 2). All data have been normalized to cycle 1 day 1 for each individual animal. * indicates P<0.05.

Results of the cytokine analysis for IL-1β, TNF-α, IL-4, and IL-10 of all pigs are displayed in Fig 1. When compared to lean pigs, obese pigs tended to show lower levels of IL-1β, TNF-α, IL-4, and IL-10, although this varied by cycle day. Obese pigs also had lower concentrations of IFN- α and IFN- γ, and higher levels of IL-8 than lean pigs, however this did not reach significance criteria (data not shown). Values were lower in obese pigs for the following cytokines: IL-1β levels between groups on day 12; IL-10 on day 1, day 16, and day 18; and IL-4 on day 8, day 12, day 16 and day 18. Concentrations had a tendency to be highest on day 12 and lowest on days 1 and 18, indicating the possibility of a cyclical effect.

### Microbial assessment

**Fig 2:**
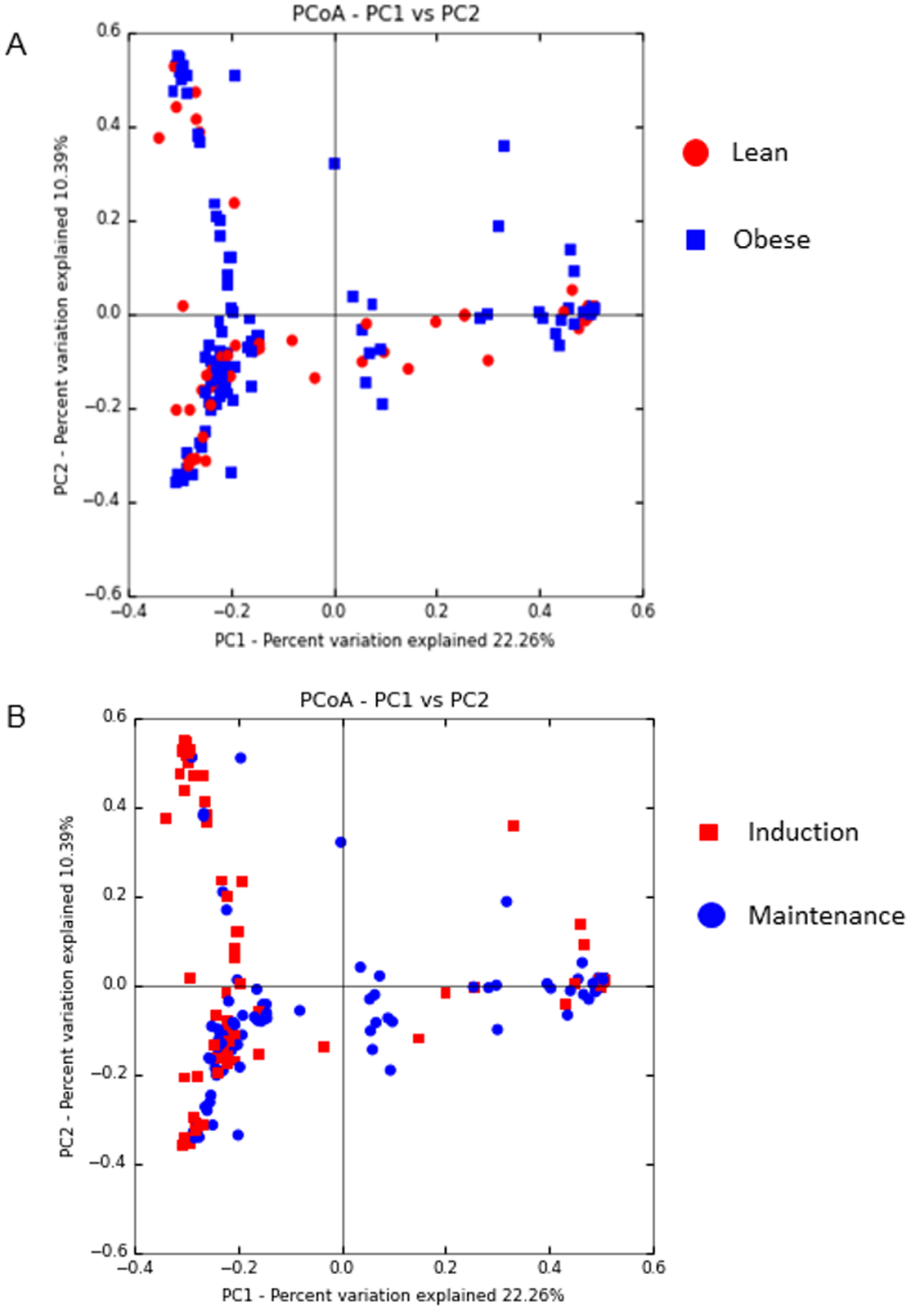
PCoA plots of distances between pig urogenital bacterial communities. Variances explained are shown on the axes. Plots include all vaginal swab and cervical flush samples taken of each pig throughout the course of the study. (A) Comparison of lean and obese animals throughout the whole study. (B) Comparison of all samples from both obese and lean animals during the induction versus the maintenance phase of the diet. Each point represents one sample. The color key provides information to determine the influence of features on spatial placement.

**Fig 3:**
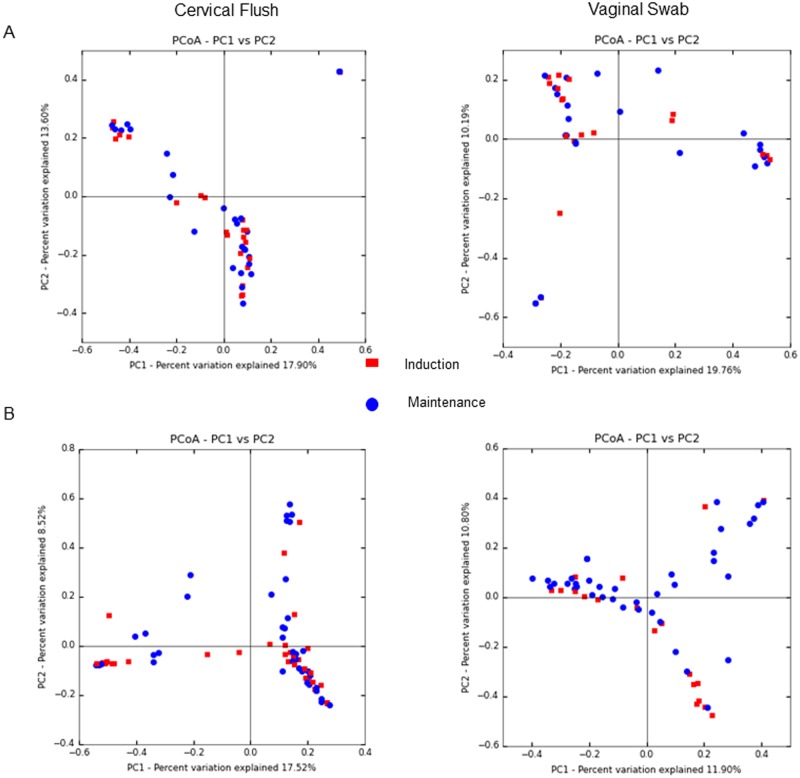
PCoA plots of distances between pig urogenital bacterial communities based on sample type and phase of diet study (A = lean and B = obese).

**Fig 4:**
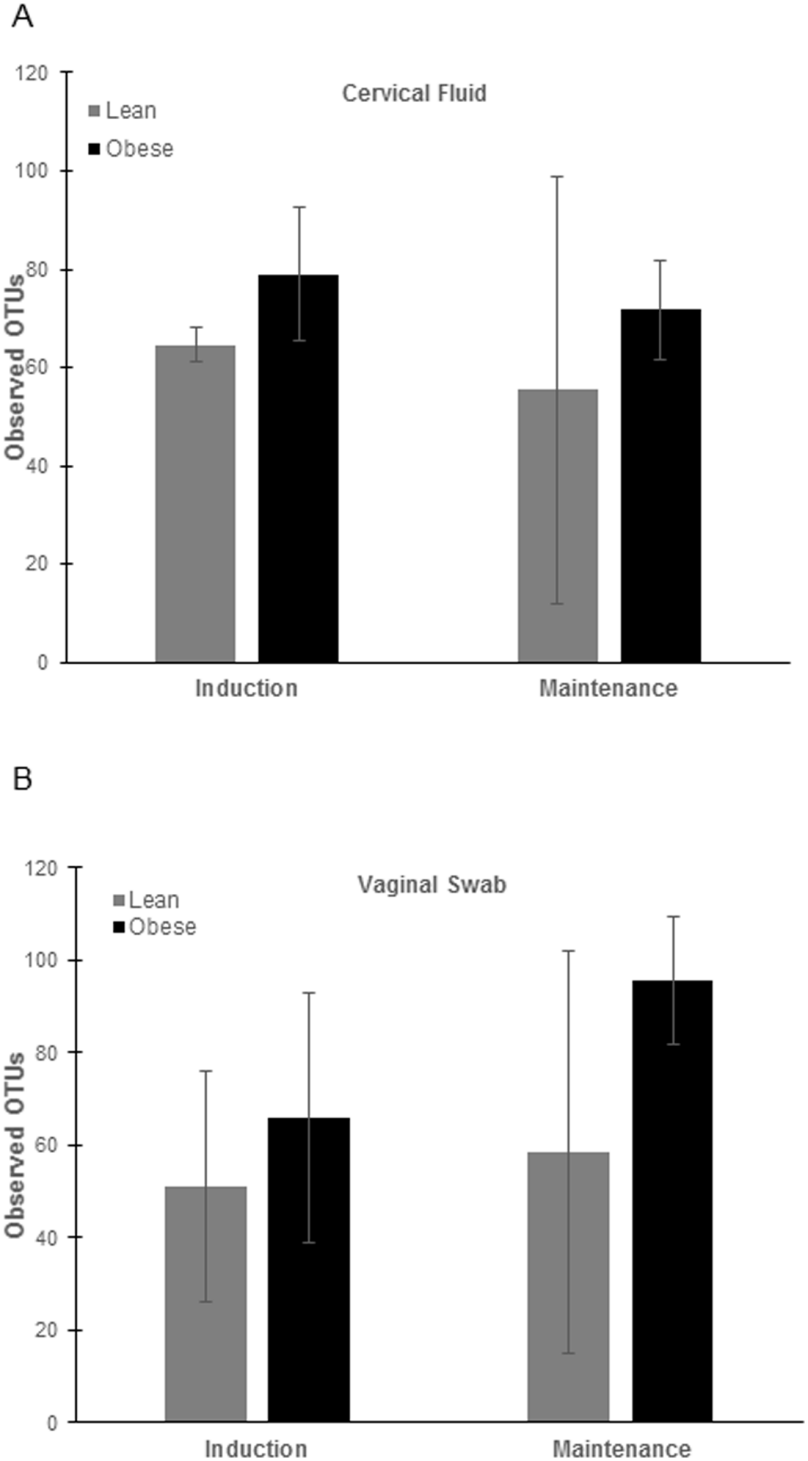
Alpha-diversity on number of OTUs observed. Results are expressed as least squared mean ± SEM (control, *n* = 3; obese, *n* = 2).

The microbial communities isolated from vaginal swabs and cervical flushes were clustered into OTUs, which were then visualized, on principal coordinates analysis (PCoA) plots (Fig 2). The axes represent 22.26% (axis 1) and 10.39% (axis 2) of the data variance. These plots demonstrate that samples clustered separately dependent on the phase of the dietary intervention (Fig 2B) and that obese animals had greater diversity in both induction and maintenance phases of the diet compared to lean animals (Fig 2A). Surprisingly we also did not observe differences in clustering patterns by stage of estrous cycle, as measured by days throughout the estrous cycle (data not shown). When analyzing vaginal swabs and cervical flushes separately, we found that in lean animals there was not a shift in microbial dynamics during different phases of the diet (Fig 3A). However, in obese animals there was a shift in microbial communities between diet induction and maintenance and that this shift was more prevalent in vaginal swabs then cervical flushes (Fig 3B). Alpha diversity measures indicated no significant difference in the number of OTUs observed (*P*>0.5; Fig 4) between obese and lean animals for either cervical fluid or vaginal swabs, indicating that the copy of expression within each bacterial taxonomy was driving diversity differences between the induction and maintenance phase of the diet period.

**Fig 5:**
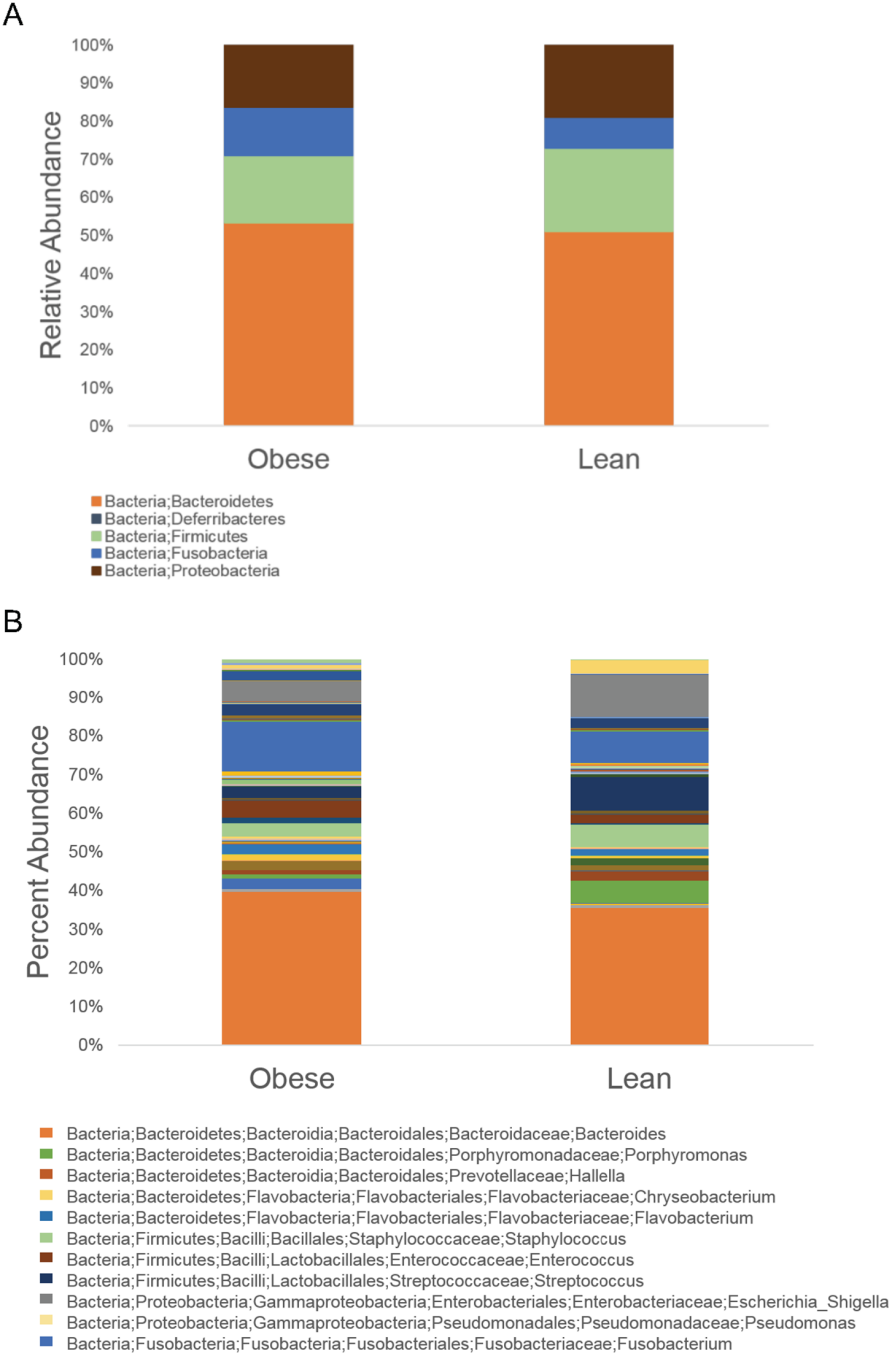
Taxon summary of relative abundance within urogenital microbial communities of control and obese pigs shown as an average of all animals from each treatment group. Taxa with the highest abundance is presented in the key. (A) Phylum-level relative abundance. (B) Genus-level relative abundance.

**Fig 6:**
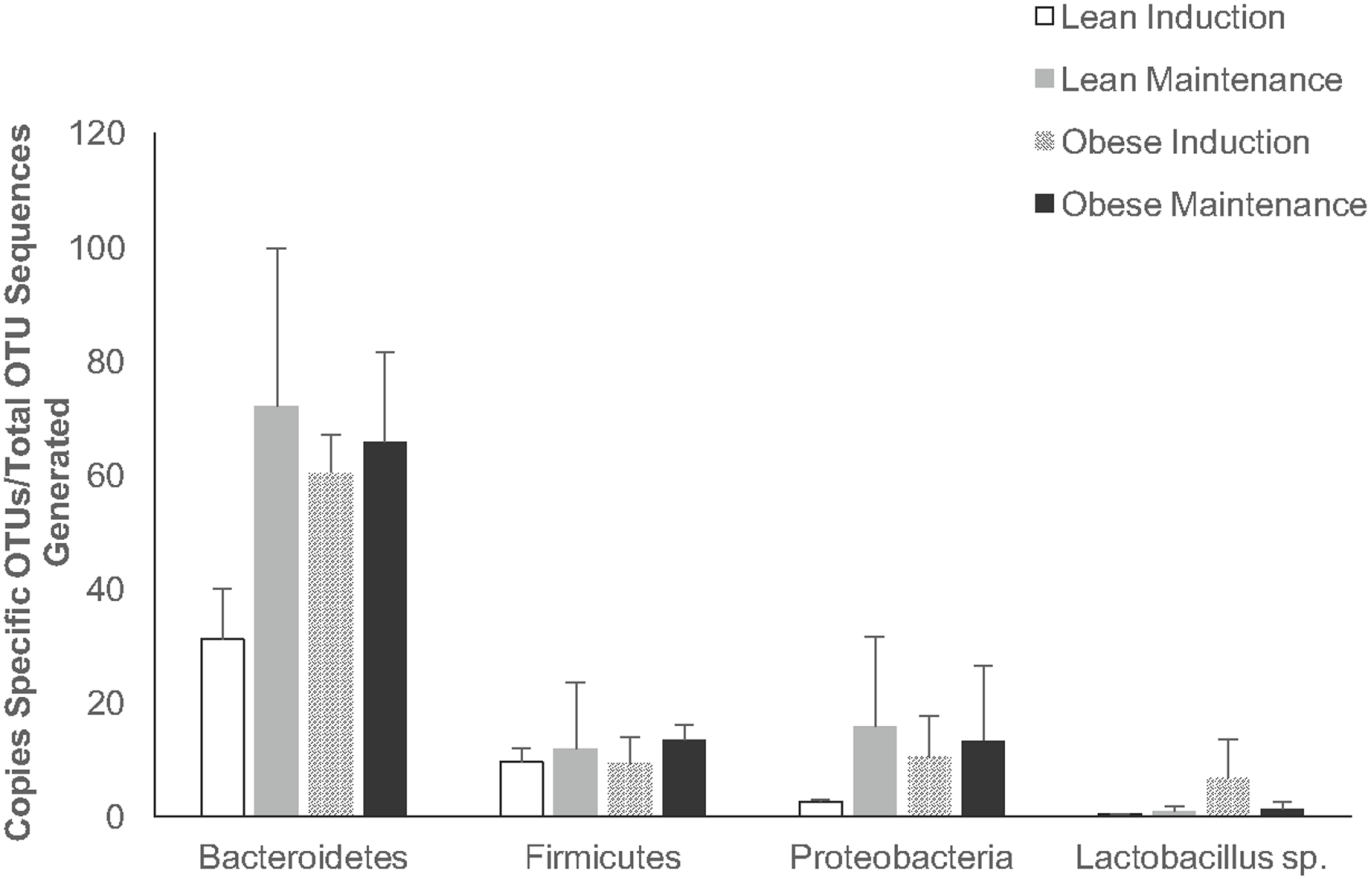
Relative abundance of prominent phyla and *Lactobaciullus* genus in vaginal swabs and cervical flushes of both treatment groups during induction and maintenance phases. Data are shown as least squares mean ± SEM.

To analyze taxonomical diversity, we measured the percent abundance of level 6 taxonomy. The three most abundant phyla across all animals were Bacteroidetes (lean, 51.07%; obese, 53.23%), Firmicutes (lean, 21.81%; obese, 17.53%), and Proteobacteria (lean, 18.93%; obese, 16.40%) (Fig 5A). The level 6 taxon summary revealed a total of 84 genera identified between the two treatment groups after sequencing and OTU clustering (Fig 5B). Seventy-six genera were identified in obese animals, whereas 64 genera were identified in lean animals. Out of the 84 total genera identified, 56 were observed in both treatment groups, 8 genera were unique to the lean group, and 20 were unique to the obese animals. The top three bacterial genera that were expressed in obese animals but not lean were Incertae_Sedis_XI;Anaerosphaera (1.11% of urogenital microbiome composition), Moraxellaceae;Acinetobacter (0.31%), and Lachnospiraceae;Blautia (0.16%). Conversely, the top bacterial genera that were identified in lean animals only were Ruminococcaceae;Oscillibacter (0.54% of urogenital microbiome composition), Lachnospiraceae;Clostridium_XlVa (0.40%), and Clostridiales_Incertae_Sedis_XII;Acidaminobacter (0.38%). The three most abundant phyla as well as the genus *Lactobacillus* were then analyzed between treatment groups during both the induction phase and maintenance phase (Fig 6). Copy number of Bacteroidetes, Firmicutes, and Proteobacteria OTUs were calculated out of total phylum OTU sequences generated. *Lactobaciulls* sp. copy numbers were calculated out of total Firmicutes OTU sequences generated. There was no statistical difference in copy number between the treatment groups.

After sequences were clustered into OTUs, bacterial taxa that either had increased abundance or decreased abundance were identified in obese animals when normalized to lean animals (Table 3). *Lachnospiracea* and *Incertae* are the genera with the highest increase in copy number in obese pigs, with a 22.47 and 18.92 fold increase compared to lean pigs, respectively. Both of these genera are associated with protective or anti-inflammatory properties [23, 24]. *Lactococcus*, *Paraprevotella*, *Streptococcus*, and *Pseudomonas* bacterial genera all have a reduced abundance in obese pigs (0.46, 0.34, 0.33, and 0.30 fold decrease, respectively). Of these, species within the genera *Paraprevotella*, *Streptococcus*, and *Pseudomonas* are associated with either inflammatory or disease states [25–27].

**Table 3:** Bacterial taxa up-regulated and down-regulated in obese compared to lean pigs.

Upregulated	
**Increased Abundance**	**Fold Increased**
Firmicutes;Clostridia;Clostridiales;Lachnospiraceae;Lachnospiracea_incertae_sedis #	22.47
Firmicutes;Clostridia;Clostridiales;Clostridiales_Incertae_Sedis_XI;Finegoldia #	18.92
Proteobacteria;Gammaproteobacteria;Pasteurellales;Pasteurellaceae;Actinobacillus *	18.20
Firmicutes;Clostridia;Clostridiales;Peptostreptococcaceae;Peptostreptococcus #	17.15
Bacteroidetes;Bacteroidia;Bacteroidales;Porphyromonadaceae;Parabacteroides #	15.91
Bacteroidetes;Flavobacteria;Flavobacteriales;Flavobacteriaceae;Wautersiella	11.83
Proteobacteria;Gammaproteobacteria;Xanthomonadales;Sinobacteraceae;Hydrocarboniphaga	7.83
Bacteroidetes;Sphingobacteria;Sphingobacteriales;Sphingobacteriaceae;Pedobacter #	5.91
Proteobacteria;Gammaproteobacteria;Xanthomonadales;Xanthomonadaceae;Stenotrophomonas *	5.19
Bacteroidetes;Bacteroidia;Bacteroidales;Porphyromonadaceae;Tannerella	4.73
Firmicutes;Clostridia;Clostridiales;Lachnospiraceae;Roseburia #	4.73
Proteobacteria;Gammaproteobacteria;Pseudomonadales;Moraxellaceae;Moraxella *	4.14
Firmicutes;Bacilli;Bacillales;Alicyclobacillaceae;Tumebacillus	4.08
Firmicutes;Bacilli;Lactobacillales;Aerococcaceae;Aerococcus	3.92
Firmicutes;Negativicutes;Selenomonadales;Veillonellaceae;Veillonella *	3.57
Proteobacteria;Alphaproteobacteria;Rhizobiales;Hyphomicrobiaceae;Gemmiger	3.40
Bacteroidetes;Sphingobacteria;Sphingobacteriales;Chitinophagaceae;Sediminibacterium	3.35
Proteobacteria;Betaproteobacteria;Burkholderiales;Comamonadaceae;Variovorax *	3.25
Firmicutes;Bacilli;Bacillales;Planococcaceae;Lysinibacillus	2.96
Proteobacteria;Alphaproteobacteria;Rhizobiales;Methylobacteriaceae;Methylobacterium	2.69
Bacteroidetes;Flavobacteria;Flavobacteriales;Flavobacteriaceae;Chryseobacterium	2.43
**Decreased Abundance**	**Fold Decreased**
Firmicutes;Bacilli;Lactobacillales;Streptococcaceae;Lactococcus #	0.46
Bacteroidetes;Bacteroidia;Bacteroidales;Prevotellaceae;Paraprevotella *	0.34
Firmicutes;Bacilli;Lactobacillales;Streptococcaceae;Streptococcus *	0.33
Proteobacteria;Gammaproteobacteria;Pseudomonadales;Pseudomonadaceae;Pseudomonas *	0.30
Bacteroidetes;Bacteroidia;Bacteroidales;Porphyromonadaceae;Paludibacter	0.19
Bacteroidetes;Bacteroidia;Bacteroidales;Porphyromonadaceae;Porphyromonas	0.18
Firmicutes;Clostridia;Clostridiales;Clostridiaceae_1;Clostridium_sensu_stricto #	0.11

*Taxon has pro-inflammatory/pathogenic properties.

^#^Taxon has anti-inflammatory/commensal properties.

## Discussion

The goal of this study was to investigate the relationship between obesity induced by a high fat diet rich in hydrogenated fats and a plant-derived saturated fat, coconut oil, on metabolism and immune and microbial parameters using the Ossabaw mini-pig as a model for obesity. Rodents are a common animal model used to study obesity; however these animals may not mimic the pathogenesis of obesity observed in humans [28]. Furthermore, a longitudinal study design would have been prohibited in a rodent model due to the required frequent sampling and the amount of sample needed per time point. Although non-human primates more closely resemble physiological parameters and disease pathology in humans, this animal model also has limitations, including increased costs to house, longer times to reach adulthood, and increased risk for zoonotic diseases. Pigs are a suitable animal model to study obesity as their anatomy, physiology, biochemical properties, lipoprotein profile, reproductive cycle length are similar to that of humans [29–31]. The major findings of this study are female Ossabaw pigs fed an excess calorie, high-fat diet, high-cholesterol, high-fructose diet rich in coconut oil: 1) developed obesity but were normoglycemic; 2) were in a state of immune homeostasis; and 3) had a greater urogenital bacterial diversity that was driven by the high fat diet.

Although coconut oil has been shown to improve glucose homeostasis [32, 33] and to improve cholesterol values in lean animals and humans, respectively, through increasing high density lipoprotein (HDL) [34], to date the effects of coconut oil on metabolic parameters in the context of obesity have not been explored. Additionally, while type II diabetes is a known sequela to obesity, the exact mechanistic cause for this relationship remains to be elucidated [35]. Recently, it has been suggested that the development of diabetes in obesity may be due to endoplasmic reticulum stress [36] or systemic inflammation [37]. In previous studies of the effects of a high fat diet on glycemic control in obese Ossabaw pigs, high fat diets that included both fructose and partially hydrogenated soybean oil but not coconut oil resulted in hyperglycemia and insulin resistance as measured by HOMA-IR in the high fat fed pigs [13, 14]. The fact that pigs in the current study that were fed a high fat diet containing coconut oil, fructose, and partially hydrogenated soybean oil became obese but do not manifest hyperglycemia and demonstrate decreased insulin concentrations while on the diet is intriguing and indicates a possible role for coconut oil in the modulation of glucose homeostasis. It is possible that the demonstrated anti-inflammatory effects of the dietary coconut oil are driving the maintenance of normal glucose control despite the presence of obesity. However, as we did not feed lean pigs a control diet supplemented with coconut oil we cannot unequivocally support this conjecture with only the current data set.

The free fatty acid profile identified increased levels of vaccenic acid and decreased levels of arachidonic acid in obese pigs compared to lean pigs. Vaccenic acid is a trans fatty acid, and may have a beneficial role in reducing risk factors of cardiovascular disease [38–40]. In contrast, arachidonic acid is a polyunsaturated fatty acid (PUFA), and a precursor for inflammatory pathways [41]. Specifically, arachidonic acid is a substrate for cyclo-oxygenase (COX) enzymes, including COX-2, an enzyme that plays a major role in the production of specific eicosanoids, called prostanoids, which are increased in inflammatory environments and act to mediate inflammation typically through G protein-coupled receptors [42]. Therefore, the free fatty acid profile of the obese pigs in the current study suggests that these animals may be more protected from inflammatory events than lean pigs are. Future studies that examine the effect of coconut oil in a control diet fed to lean pigs are warranted to fully assess the potential beneficial effect of coconut oil on inflammation and immune function.

Identification of bacterial taxa that have either increased or decreased abundance in obese compared to lean pigs in the urogenital tract revealed that obese pigs were more protected from inflammation within the urogenital tract. These data are novel as the influence of obesity on urogenital microbiome community dynamics has never been reported but the influence of obesity on gut microbial function and inflammation has been well established [43]. Therefore, we can utilize what is already known regarding the function and abundance of specific taxa in the gut inflammatory phenotype and extrapolate this information to the urogenital tract. As mentioned above, the genera *Lachnospiracea* and *Incertae* have the highest increase in copy number in obese pigs and are associated with protective or anti-inflammatory properties. *Lachnospiracea* species are decreased in the gut microbiome in patients with inflammatory bowel disease [44], and have also been shown to exhibit colonization resistance against pathogenic *C*. *difficile* [23]. *Incertae* species within the gut are associated with reduced intestinal inflammation in patients with Crohn’s disease [45]. Furthermore, Bajaj et al. observed a lower abundance of *Incertae* species within the gut in patients with cirrhosis, and also that a less robust immune response is initiated against these species, concluding that the presence of these bacteria are associated with decreased inflammation [24]. Conversely, *Paraprevotella*, *Streptococcus* and *Pseudomonas*, which have reduced abundance in the urogenital tract of obese pigs when compared to lean pigs, contain species that are linked to inflammatory and/or disease states. *Paraprevotella* is closely related to *Prevotella* species, which are associated with the pathogenesis of rheumatoid arthritis [46]. The inflammatory pathway induced by *Streptococcus pneumoniae* infection, the causative agent for bacterial meningitis, is well known [47]. The genus *Pseudomonas* contains the species *Pseudomonas aeruginosa*, the established infectious agent in cystic fibrosis patients that results in a strong inflammatory response [48]. Interestingly, obese pigs also had reduced abundance of *Lactococcus* species within the urogenital tract, which are lactic-acid producing commensal bacteria. Lactic acid serves to protect the vaginal canal by several mechanisms, including lowering the pH of the vaginal canal, ultimately creating an environment that disallows pathogenic bacteria from flourishing and causing infection [49]. However, the exact role of the species within the genus *Lactococcus* in the female urogenital tract is not as well characterized as *Lactobacillus* species, warranting further investigation on the extent of the protective effects *Lactococcus* offers.

Obese pigs had reduced serum levels of both pro-inflammatory and anti-inflammatory cytokines compared to lean animals. Increased TNF-α concentrations from adipose tissue are typically observed in obese individuals, and are associated with insulin resistance [50, 51]. However, in the current study we found that both pro-inflammatory cytokines (specifically IL-1β and TNF-α) and anti-inflammatory cytokines (IL-10 and IL-4) were down-regulated in these obese Ossabaw pigs compared to lean pigs. Based on our results we hypothesize that the obese pigs fed at high fat diet with coconut oil are in a state of immune homeostasis and have more conferred immune protection from pro-inflammatory events than lean pigs fed a control diet.

The microbial composition of the gut is associated with obesity perhaps by influencing metabolic function, gut permeability, and inflammation [50–53]. A second focus for this study was on the relationship between reproductive function and inflammation by means of investigating the urogenital microbiome. Bacterial diversity is described as the number and abundance of specific taxa within a given environment, and balance of diversity is crucial to both protect against a pathogen breach and also maintain a stable, healthy environment. Low microbial diversity is associated with conditions such as obesity and inflammatory bowel disease [54, 55]. On the other hand, bacterial vaginosis (BV) exhibits a high bacterial diversity. Fredricks et al. characterized the microbial environment of women with BV as complex and containing a variety of infection-causing bacterial species specific to this disease [56]. Therefore, it is generally accepted that a moderately diverse microbiome confers the most protection from infection. In the current study, obese pigs had an increased bacterial diversity in the urogenital tract as indicated by the PCoA plot. The increase in copy number of protective bacterial species in obese pigs suggests that the diversity of bacterial species in these animals had a protective effect from pathogenic insult. The PCoA data also suggests that bacterial diversity in the urogenital tracts of obese pigs was driven by a high fat diet, as diversity increased once the animals reached the maintenance phase. This finding is underscored by the fact that there were no clustering patterns observed in the PCoA plots when looking at either sample type or day within the estrous cycle. The data from this study indicate that there may be a distinct microbiome “signature” associated with obesity. While this has already been studied in humans [51, 57], our experimental model has the advantage of utilizing animals of the same genetic background, allowing us to study the effects of dietary intervention with minimization of external variables. Thus, our study was more targeted in identifying a signature in obesity associated with diet manipulation specifically.

The fact that obese pigs fed a high fat diet rich in coconut oil have decreased inflammation and levels of arachidonic acid is intriguing, since obesity alone is known to drive inflammation. One key difference between the high fat in this study, the high fat diet used in previous Ossabaw studies by Newell-Fugate et. al., and the lean diet was the inclusion of coconut oil in the high fat diet for this current study [13, 14]. Coconut oil contains medium-chain fatty acids and has been shown to have anti-inflammatory properties, including the ability to decrease pro-inflammatory cytokines *in vivo* [58, 59]. Additionally, several studies have described the effects of coconut oil on methanogenesis in rumen fermentation, and also on volatile fatty acid production by bacterial species within the rumen [60, 61]. It has also been shown that supplementation of coconut oil in the diet increases overall bacterial counts in the rumen [60]. More interesting, perhaps, are the antimicrobial properties of lauric acid, which is present in high amounts in coconut oil. Wang et al. described how the antimicrobial properties of lauric acid inhibited several pathogenic bacterial species such as *Listeria monocytogenes* [62]. Based on our results, we postulate that coconut oil in the high fat diet might modulate the peripheral immune system, possibly through regulation of microbial community dynamics.

In conclusion, when fed an excess-calorie, high-fat/cholesterol/fructose diet rich in coconut oil, female Ossabaw pigs developed obesity but were normoglycemic, demonstrated decreased markers of inflammation, and had greater bacterial diversity in the urogenital tracts than lean pigs. Obesity has been implicated as a major cause of type II diabetes and infertility. However, our results indicate that obesity alone may not be solely responsible for these pathologies. Instead, dietary fat source may modulate glucose homeostasis in the context of obesity, possibly secondarily to the inflammatory environment. Our results suggest that dietary modifications may be beneficial to modulate obesity to a “healthy phenotype”. Because the development of obesity is multifactorial, the Ossabaw pig is a useful animal model in which to study the underlying mechanisms of this condition and the influence of diet within the context of obesity.
